# Guidelines from the expert advisory committee on the Safety of Blood, Tissues and Organs (SaBTO) on patient consent and shared decision‐making for blood transfusion

**DOI:** 10.1111/bjh.70075

**Published:** 2025-09-09

**Authors:** Michael F. Murphy, Damien Carson, Anwen Davies, Stephanie Ditcham, Graham Donald, Roger Graham, Lizzie Hutchinson, Paul Kerr, Denise McKeown, John Moppett, Shruthi Narayan, Paolo Polzella, Ella Poppitt, Megan Rowley, Tracey Shackleton, Julie Staves, James Neuberger

**Affiliations:** ^1^ NHS Blood & Transplant Oxford UK; ^2^ Oxford University Hospitals NHS Foundation Trust Oxford UK; ^3^ University of Oxford Oxford UK; ^4^ Ulster Hospital Dundonald UK; ^5^ Welsh Blood Service Pontyclun UK; ^6^ Royal Devon University Healthcare NHS Foundation Trust Exeter UK; ^7^ Royal Victoria Hospital Belfast Belfast UK; ^8^ Bristol Royal Hospital for Children Bristol UK; ^9^ Scottish National Blood Service Edinburgh UK; ^10^ Alder Hey Hospital Liverpool UK; ^11^ SaBTO London UK

**Keywords:** blood transfusion, consent to transfusion, patient information, refusal of transfusion

## Abstract

Evidence from national audits of practice indicates that the provision of information to patients about transfusion and the taking of consent to transfusion have not improved in recent years. Although the final report of the Infected Blood Inquiry did not make a specific recommendation about consent to transfusion, it emphasised the need for cultural change, the importance of openness and giving patients a voice. The purpose of these updated Safety of Blood, Tissues and Organs (SaBTO) guidelines is to enhance the provision of information to patients about blood transfusion, ensure an effective process for obtaining patients' consent and support shared decision‐making. The guidelines emphasise that there is a duty on staff administering a transfusion to check that the documentation for consent is present and valid before commencing the transfusion. Hospitals and other healthcare facilities must facilitate this step by ensuring that documentation for consent can be easily found in a standard format and location in the patient's paper or preferably their electronic record. They should employ mechanisms through their arrangements for clinical governance to support the training of all staff who may take consent to transfusion and monitor the implementation and compliance with these SaBTO recommendations with subsequent improvement plans developed and implemented if necessary.

## Graphical abstract

The key points to be discussed with a patient when obtaining consent to transfusion are the reason for the transfusion, the benefits and risks of transfusion, the possible alternatives to transfusion, the process for transfusion, any specific transfusion requirements that the patient may have, the consequences of refusing transfusion and the ability to withdraw consent to transfusion at any time, that recipients of transfusion are unable to donate blood in the United Kingdom and the encouragement to patients to ask any questions they may have about transfusion.

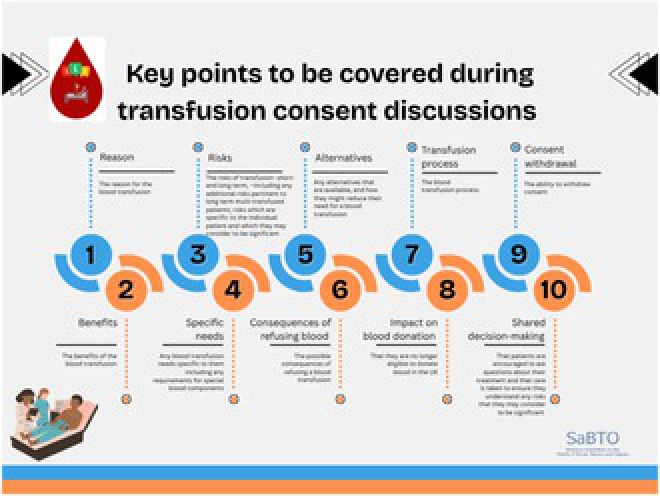

## METHODOLOGY

These guidelines were prepared by an expert Safety of Blood, Tissues and Organs (SaBTO) working group and update the previous SaBTO Patient Consent to Blood Transfusion Guidelines (2020). The members of the working group were selected by the Chair of the Working Group and the SaBTO Chair to provide medical, nursing and scientific expertise and involvement from lay members.

### Literature review details

The PubMed database was searched for the English language articles with abstracts from 2020 (date of publication of previous SaBTO guidance on this topic) to 2025 using the following search terms: ‘consent to transfusion’; ‘patient information for transfusion’; and ‘refusal of consent to transfusion’. References from relevant publications were also searched. It was not considered that the search yield provided any recent primary literature to guide the working group recommendations.

### Review of the manuscript

This was performed by members of SaBTO and the manuscript was approved on 3 June 2025.

## INTRODUCTION AND BACKGROUND

The need to update the report of the 2020 Advisory Committee on the SaBTO entitled *Blood Transfusion: Patient Consent* ^1^ was identified by the Chair of SaBTO. It was considered timely to do this as the Infected Blood Inquiry report^2^ identified concerns about whether and to what extent people were treated with blood transfusion without their full knowledge or consent. In November 2024, a SaBTO consent to blood transfusion working group was established. The remit and scope of this group, approved by SaBTO, which advises UK ministers and health departments on the most appropriate ways to ensure the safety of blood, cells, tissues and organs for transfusion or transplantation, included the following:

- To assess the value provided by the *2020 Patient Consent to Blood Transfusion* ^1^ guidance to determine whether there is a need to update, maintain or withdraw the guidelines, in part or whole.
- To review the implementation of the guidance, for example, through the annual audits of compliance with the standard on patient information for blood transfusion.
- To make recommendations on how to improve the implementation of the guidance by providing examples of good practice, for example, on how consent to blood transfusion should be documented, and the training of relevant staff.
- To make recommendations on obtaining consent for neonates, children and young adults; the 2020 SaBTO guidance did not provide recommendations for consent to transfusion in children.
- ‘Blood transfusion’ for the purposes of this working group refers to the transfusion of blood components as a therapeutic constituent of blood [red blood cells, platelets, fresh‐frozen plasma (FFP), cryoprecipitate and granulocytes]. Although technically a blood product, for the purpose of consent to blood transfusion, solvent detergent (SD) FFP should be subject to the same consent processes and recommendations as blood components.
- Blood products, that are therapeutic materials derived from blood or plasma, such as coagulation factor concentrates, human albumin solution, intravenous immunoglobulin and specific immunoglobulins of human origin such as anti‐D immunoglobulin) are classified as medicinal products and are subject to different regulations. Blood products as medicines will have an accompanying specific patient information leaflet (PIL), which provides information on using the medicine safely and the product's properties and side effects. However, as these are of human origin, the same principles apply for obtaining consent to the transfusion of blood products as those outlined in this guidance document for blood components, even if the products have been treated to minimise the risk of transfusion‐transmitted infection and the risks of some other complications of transfusion are lower.
- Individuals who have received a transfusion of a blood component in the UK from 1st January 1980 are excluded from blood donation because of concerns about transmission of variant Creutzfeldt‐Jakob dsease. This exclusion also applies to any individuals who have been treated with blood‐derived coagulation factor concentrates, prothrombin complex concentrates, subcutaneous or intravenous immunoglobulin or who have undergone plasma exchange. If treatment with specific immunoglobulin has been given as prophylaxis such as anti‐D, anti‐tetanus or hepatitis immunoglobulin, blood donation is permitted.
- The recommendations are pertinent to patients who may be exposed to blood components and blood products in any healthcare setting, including, for example, patients undergoing extracorporeal membrane oxygenation (ECMO), exchange transfusions and pump priming for organ perfusion. They also apply to autologous (obtained from the same individual) as well as allogeneic (donated) transfusions as many of the most frequent serious risks of transfusion are similar (e.g. transfusion‐associated circulatory overload [TACO] and wrong blood component transfused).

The recommendations, which are summarised in Appendix S1, are aimed to ensure that patients are informed about blood transfusion and are enabled and empowered to discuss their treatment options. They are a set of principles which should be incorporated into local practices for all patients, taking into account specific issues related to children and those with deemed mental incapacity. It is not the remit of these recommendations to provide detailed guidance related to reduced mental capacity, refusal of blood components (including advance directives) or to advise on legalities related to consent which should be covered by standard hospital practices.

**Recommendation**

- **The purpose of these updated recommendations is to enhance the provision of pertinent information to patients about blood transfusion, ensure an effective process for obtaining patients' consent to blood transfusion and support shared decision‐making. The recommendations are pertinent to patients who may be exposed to blood components and blood products in any healthcare setting**.

### Considerations by the working group

The working group members were selected to represent a broad range of experiences of transfusion practice and included doctors, nurses, scientists and patients. The working group met on four occasions and corresponded by email to finalise the guidelines.

The working group took into account the 2015 decision of the UK Supreme Court in Montgomery *v* Lanarkshire (UKSC 2013/0136).^3^ This was a landmark legal decision for informed consent and the shared decision‐making model practiced in the United Kingdom. The court's decision redefined the standard for informed consent and disclosure. The Supreme Court held that a patient should be told whatever they wanted to know, not what the doctor thinks they should be told, and established a duty of care to warn of material risks.

The test of materiality defined in the Montgomery ruling was whether ‘a reasonable person in the patient's circumstances would be likely to attach significance to the risk, or the doctor is or should reasonably be aware that the particular patient would be likely to attach significance to it’. The doctor is therefore under a duty to take reasonable care to ensure that the patient is aware of any material risks involved in any recommended treatment and of any reasonable alternative or variant treatments. This clarifies that, when seeking consent to treatment, the question of whether the information given to the patient is adequate is judged from the perspective of a reasonable person in the patient's position. For the purposes of consent, the ruling from Montgomery established a duty of care to warn of material risks and the patient's right to make informed treatment decisions takes precedence above the healthcare practitioner's professional judgement/discretion in disclosing information. It represents a shift towards a more collaborative approach to consent between patients and medical practitioners. This means finding the time to explain the risks and benefits of a recommended course of action as well as the other options. The ruling makes it clear that any intervention must be based on a shared decision‐making process, to help patients make an informed choice.

The working group also considered the National Institute for Health and Clinical Excellence (NICE) Blood Transfusion guideline (NG24) (2015)^4^ and the NICE Blood Transfusion Quality Standard (2016),^5^ which both include the provision of verbal and written information about blood transfusion and its benefits, risks, alternatives and implications. In 2024, the National Comparative Audit of compliance^6^ with the NICE Quality Standards for Transfusion found that the implementation of informed consent to transfusion was sporadic and compliance was generally low; only 36% of patients had documentation that they were given verbal and written information about blood transfusion. This was only marginally improved from 35% in the 2023 audit and only slightly improved from 26% in the 2021 audit.

The working group also considered examples of poor consent and shared decision‐making practices in reports submitted to the Serious Hazards of Transfusion (SHOT).^7^

Anecdotal evidence and the experiences of the SaBTO consent working group members suggest that current practice has not significantly improved in recent years. Although the final report of the Infected Blood Inquiry did not make a specific recommendation about consent to blood transfusion, it emphasised the need for cultural change, the importance of openness and giving patients a voice.^2^

## INFORMED AND VALID CONSENT

Historically, it was the remit of a doctor to undertake the consent process. As non‐medical roles have developed and advanced, a wider range of healthcare practitioners are now involved in consent. These healthcare practitioners should be trained and deemed competent (as per local hospital policy) to seek patient consent, be familiar with the key principles of good practice in obtaining it and have sufficient knowledge and experience of blood transfusion to be able to provide the information needed for the patient to make a decision, answer any questions that may be raised and be aware of the range of ethical issues that commonly arise in transfusion practice. The provision of information and the informed consent discussion should be undertaken prior to a decision to transfuse, wherever possible.

Informed and valid consent is the process by which a patient learns about and understands the purpose, benefits and potential risks of the blood transfusion. For consent to be valid, it must be voluntary, informed and given by a competent patient with capacity.^8^, ^9^ Consideration should be given to whether the blood transfusion is the only available treatment, whether any reasonable alternative treatments are available, and the comparative risks and benefits of those alternatives.^10^ In addition to the provision of information about the nature and purpose of the proposed treatment, an active discussion should result in shared decision‐making. The patient should be encouraged to ask their own questions and to raise any concerns they wish to be addressed before they make a decision to receive or refuse the blood transfusion. The dialogue needs to be focused on the individual to ascertain what risks are or are not acceptable to that individual's circumstances. Non‐medical considerations may influence a patient's choice. What is not a material risk for one patient may be a material risk to another. The healthcare practitioner must provide information in a comprehensible way and ensure it is understood. The detail desired varies from patient to patient; the information required to make consent informed may vary depending on the complexity and risks of treatment as well as the patient's wishes.

**Recommendations**

- **Informed and valid consent to blood transfusion must be obtained for all patients who may, are likely to, or will definitely receive a blood transfusion. This includes where blood transfusion might be needed at some later time, for example, during surgery when the patient is incapacitated. An indication that blood transfusion may be required is the collection of a ‘group and save’ or ‘crossmatch’ sample**.
- **Hospitals must have policies that cover the provision of information about blood transfusion and for obtaining patients' consent to blood transfusion. The policies must include the processes that healthcare staff should follow and how they should be documented**.
- **Consideration must be given whether the blood transfusion is the only available treatment, whether any alternative treatments are available and suitable, and the risks and benefits of those alternatives to blood transfusion. An active discussion must result in shared decision‐making, allowing the patient to ask their own questions and to raise any concerns that they wish to be addressed before they decide to receive, or refuse, the blood transfusion. Such shared decision‐making discussions must be documented in the patient's clinical record, ideally electronically**.
- **The following framework (adapted from the NICE Blood Transfusion Guideline 2015 NG24** ^4^ **and taking into account the ruling from Montgomery** ^3^ **establishing a duty of care to warn of material risks and the patient's right to make informed treatment decisions) should be used when providing verbal and written information to patients and their family members or carers (as appropriate):**
  – **The reason for the blood transfusion;**
  – **The benefits of the blood transfusion;**
  – **The risks of transfusion—short and long term, including any additional risks pertinent to long‐term multitransfused patients; risks which are specific to the individual patient and which they may consider to be significant;**
  – **Any blood transfusion needs specific to them including any requirements for special blood components;**
  – **Any alternatives that are available, and how they might reduce their need for a blood transfusion;**
  – **The possible consequences of refusing a blood transfusion;**
  – **The blood transfusion process;**
  – **That they are no longer eligible to donate blood in the United Kingdom;**
  – **The ability to withdraw consent;**
  – **That patients are encouraged to ask questions about their treatment and that care is taken to ensure they understand any risks that they may consider to be significant**.

### Refusal of consent

Following discussions regarding blood transfusion as outlined above, patients may not wish to receive a blood transfusion. Such a decision may relate to all types of blood transfusion or to specific blood components or blood products. For example, a patient may be unwilling to receive a red blood cell transfusion but would consent to receive blood products. It is an important principle of consent that an adult with capacity can make decisions that the clinical team may not consider to be in the patient's best interest.

**Recommendation**

- **Patients are entitled to refuse or withdraw their consent to blood transfusion. This must be documented and managed appropriately. If the patient consents to certain blood components and/or products but not others, this must be documented in both the patient's clinical and blood transfusion laboratory records and must be easily accessible to treating teams**.

## INFORMATION AFTER TRANSFUSION (RETROSPECTIVE INFORMATION)

There are a few exceptions when treatment may be able to go ahead without the person's consent, even if they are capable of giving their permission. National Health Service (NHS) Guidance^11^ states that it may not be necessary to obtain consent if a person:

- Needs emergency treatment to save their life, but they are incapacitated (e.g. they are unconscious). The reasons why treatment was necessary should be fully explained once they have recovered and documented in the same way.
- Where there is an unexpected need for transfusion, for example, where the patient did not have a group and save before minor/moderate surgery but major blood loss occurred.

In these circumstances, information about transfusion should be provided after the patient has recovered from emergency treatment.

Where patients are deemed to lack capacity, or should a patient need to receive a transfusion in an emergency and is unable to provide consent, this must be documented in the patient's clinical record. Local procedures must be followed, including the appropriate management of patients where there is evidence that they would refuse a blood transfusion. The patient will need to be informed post‐emergency when the patient is deemed to have capacity and retrospective patient information related to blood transfusion provided. The provision of retrospective information is important to ensure not only that patients are informed of any associated potential risks relating to blood transfusion but also to ensure that they are aware that they have received a blood transfusion.

**Recommendations**

- **Patients who have received a blood transfusion and who were not able to give informed and valid consent prior to the blood transfusion must be informed of the blood transfusion in a face‐to‐face (in person) discussion. This must be documented in the patient's clinical record and in the hospital discharge summary**.
- **Patients undergoing procedures where they were told that they may or may not need a blood transfusion must be informed whether they had a blood transfusion or not. This should be documented in the patient's clinical record and in the hospital discharge summary**.

## CONSENT TO TRANSFUSION IN NEONATES, CHILDREN AND YOUNG ADULTS

The requirements for obtaining valid consent in neonates, children and young adults are well documented.^12^, ^13^ Those aged 16 or over are generally considered competent to consent to treatment including the administration of a blood transfusion.^14^ Those under 16 would in general require someone with parental responsibility (as defined by the Children Act, 1989)^15^ to consent for them except when a child under the age of 16 is considered Gillick competent.^16^ Even in that context, it is considered good practice to inform those with parental responsibility. Gillick competence is an issue‐specific assessment and depends on both the maturity and understanding of the child, and the risk and complexity of the proposed treatment.

Although the clinical context may be different, the risks and benefits of blood transfusion in neonates and children are the same as those in adults, and the same principles apply. It is therefore essential that written, verbal or electronic information is given to those with parental responsibility to enable them to make appropriate decisions. This information should focus on the specific context of blood transfusion in the paediatric population.

It is equally important, and a key principle in the care of children, that healthcare practitioners should involve children and young people in decisions about their healthcare in ways that are appropriate to their maturity and understanding and young people's experience of healthcare.^17^ Thus, age‐appropriate information about blood transfusion should be available.^18^

The one particular difference between the administration of blood components in children and young people aged 16–17 years compared to adults is the consequence of refusing consent. If those with parental responsibility or a young person aged 16–17 refuse to give consent to a particular treatment, under the Children Act (1989),^15^ this decision can be overruled by the courts if treatment is thought to be in the best interests of the child. The legal situation is complex and differs between the nations of the United Kingdom. Consultation with hospitals' legal teams is recommended at the earliest opportunity. Every effort must be made to understand the reasons for refusal of consent, appropriate alternatives considered and the views of the patient and those with parental responsibility clearly documented. In an emergency, where treatment is vital and waiting for parental consent would place the child at risk, treatment can proceed without consent.

**Recommendations**

- **Hospitals must have policies that cover consent to blood transfusion for neonates, children and young adults and also for the refusal of consent to blood transfusion in both children and adults**.
- **Children and young adults should be involved in the consent to transfusion process according to their maturity and understanding, and age‐appropriate information should be provided to support them**.
- **Consultation with hospitals' legal teams is recommended whenever there are any concerns about the process for consent to blood transfusion in neonates, children and young adults**.

## DURATION OF CONSENT

There are too many variables and individual patient scenarios for SaBTO to provide definitive guidance for all of them. The duration of consent must be discussed and agreed with the patient as part of the shared decision‐making process and in line with local policies. It is recognised that there is a difference between a patient who receives regular blood transfusions every few weeks for that condition (e.g. haemoglobinopathy) and a patient with cancer who has surgery, then a course of chemotherapy and then further surgery, with each treatment stage potentially requiring transfusion. If it is deemed appropriate that consent may span more than one blood transfusion episode, or across the duration of a patient admission period, this should be documented in the patient's clinical notes. Hospitals may consider having different policies for consent to specific groups of patients such as patients having regular blood transfusions.

Where long‐term blood transfusions are required to manage a specific condition, full and informed consent, which includes long‐term effects of blood transfusion, should be obtained at the start of their treatment plan. It is not necessary, or practical, to continue to obtain full and informed consent prior to each and every blood transfusion episode, but it is important that patients receive ongoing information regarding the risks, benefits and any potential alternatives to blood transfusion. Consent should be formally renewed if the patient raises any concerns or expresses a wish to review consent, or if new information has become available, for example, about the risks of blood transfusion or any other treatment options. Long‐term patient treatment plans should include the management of any complications of blood transfusion, and these management plans should incorporate patient consent as appropriate.

A template for the shared decision process for consent to transfusion in different clinical scenarios is shown in Appendix S2.

**Recommendations**

- **The duration of consent must be discussed and agreed with the patient as part of the shared decision‐making process. If it is deemed appropriate that consent may span more than one blood transfusion episode, or across the duration of a patient admission period, this must be documented in the patient's clinical record**.

## DOCUMENTATION OF CONSENT

It is important to recognise that seeking and obtaining of consent is more than a signature on a form. It is the process of providing the information that enables the patient to understand (and in some cases accept) risk and make a decision to receive a transfusion. This was found in the [recent] case of Thefaut v Johnston [2017] EWHC 497^19^ where Justice Green observed that: ‘It is accepted that the simple fact that Mrs Thefaut signed the hospital consent form is not to be taken as an indication of acceptance of risk. In my view the document is of no real significance on the present facts. (It would have greater significance in emergency cases involving no prior contact between patient and the clinician)’.

The 2020 SaBTO Consent to Transfusion recommendations^1^ did not require signed consent by the patient. The emphasis was on the shared evidence‐based dialogue and decision‐making element of the consent process, rather than on obtaining the patient's signature. This recommendation has not been changed, but individual organisations may choose to implement consent signed by the patient. Where consent forms include a ‘tick box or boxes’ to prompt healthcare staff, these should be formatted in a way which supports valid and informed consent, for example, by including the points in the framework for obtaining consent to blood transfusion provided above and providing a free text option for documentation of any additional discussion or concerns.

Where patients are alert and orientated, the healthcare practitioner administering the blood transfusion should confirm that valid consent has been obtained from the patient and documented prior to the administration of the blood transfusion. Healthcare practitioners should be mindful that patients can change their mind at any point, and patients are entitled to withdraw their previous consent.

Examples of methods to document consent to transfusion from the United Kingdom are shown in Appendix S3.

**Recommendations**

- **There is a duty on staff administering a blood transfusion to check that that documentation for consent to blood transfusion is present and valid before commencing the transfusion. Hospitals must facilitate this step by ensuring that documentation for consent to blood transfusion can be easily found in an agreed standard format and location in the patient's paper or electronic record**.
- **All patients who have received a blood transfusion must be provided with details of the blood transfusion together with information about any adverse events associated with the blood transfusion. Patients must also be informed that they are no longer eligible to donate blood in the United Kingdom. All relevant information must be documented in the patient's clinical record, ideally electronically and included in their hospital discharge summary to ensure their family doctor is also aware**.
- **Hospitals who already have electronic patient records must ensure the digital documentation of informed and valid patient consent to blood transfusion. Those hospitals who do not already have electronic patient records must ensure the inclusion of the digital documentation of informed and valid patient consent to blood transfusion in their plans for electronic patient records**.

## INFORMATION RESOURCES FOR PATIENTS AND PUBLIC

Providing written information to patients can help assist the consent process by giving them the opportunity to digest, recapitulate and reaffirm their decision. Patient information leaflets (PILs) which summarise the main risks and benefits of blood transfusion can help patients understand and recall this information. Such information leaflets can only provide generic information and do not take into account individual patient circumstances, conditions, values or priorities. The leaflets are intended only to support and reinforce verbal information and discussion and not to replace them.

PILs are freely available from each of the UK Blood Services. The UK Blood Services have developed a standardised PIL to ensure consistency of the information across the whole of the United Kingdom and have also provided additional online information resources for patients and the wider public.^18^

**Recommendation**

- **The UK Blood Services should continue to provide a standardised source of information for patients who may receive a blood transfusion in the United Kingdom**.

Where other organisations provide information related to transfusion (e.g. NHS Choices, or patient support organisations such as sickle cell, thalassaemia or other haematology support groups), these organisations should co‐operate with the UK Blood Services to ensure relevant up‐to‐date information is included.

## TRAINING AND INFORMATION RESOURCES FOR HEALTHCARE PRACTITIONERS

In order to provide informed and valid consent to blood transfusion, it is vital that all healthcare practitioners involved in the blood transfusion process are supported to maintain their knowledge of consent and its relevance and importance in blood transfusion.

The British Society for Haematology (2017) recommends that all staff should receive regular (minimum three yearly) knowledge and skills training in blood transfusion for all of the processes they are involved in.^20^ However, information available from the e‐learning modules in Learn Blood Transfusion and Blood Transfusion Training does not show full compliance and uptake of the specific consent modules is lower than the more generic modules.^21^

The requirement for shared decision‐making and obtaining consent to blood transfusion should be included in the training curricula and programmes of training for all relevant healthcare practitioners. This includes undergraduate training for nurses and doctors, foundation programme training and speciality training for doctors. While valid and informed consent is included in the curricula for foundation training^22^ and specialty training for doctors,^23^, ^24^, ^25^ the requirement for consent to blood transfusion is neither specifically mentioned nor assessed. Inclusion of consent to blood transfusion when curricula are updated and introduction of assessment of practice in medical training would be significant advances in raising the importance of shared decision‐making and consent to blood transfusion. This is particularly important in the light of the recommendation from the Infected Blood Inquiry that bodies responsible for undergraduate and postgraduate training ensure adequate training in blood transfusion as well as incorporating lessons learned from the Infected Blood Inquiry into every doctor's training.^2^

Training resources to support shared decision‐making and consent to blood transfusion should align with the General Medical Council (GMC) and Nursing and Midwifery Council (NMC) principles and contain the specific elements related to blood transfusion consent as detailed in this guidance. In addition, information given in evidence to the Infected Blood Inquiry should be included to exemplify the important learning lessons from the inquiry.

Healthcare professionals involved in taking consent to blood transfusion should complete the consent training relevant to their role and demonstrate they have the skill and knowledge to undertake this role by completing an assessment of competency. Consent to blood transfusion training should be completed at least every 3 years.

The evidence of completion of consent training is a professional responsibility and should be recorded in training portfolios and/or training records for each individual. These records should be auditable and compliance monitored by the NHS organisation. Employers should accept training records/certificates of completion from employment within another organisation when healthcare staff move to different roles or a different organisation within the NHS.

An accessible and up‐to‐date UK‐wide information resource for healthcare practitioners should be maintained to facilitate discussions about consent to transfusion, indicating the key issues to be discussed when obtaining informed and valid consent to a blood transfusion and providing information about the risks related to transfusion.

Training resources and additional information to support shared decision‐making and consent to transfusion are shown in Appendix S4.

**Recommendations**

- **Training and competency assessment for taking consent to blood transfusion must be included in programmes of training for all relevant healthcare practitioners, and this must be renewed every 3 years**.
- **A centralised UK‐wide information resource for healthcare practitioners should continue to be provided to support engagement with patients and facilitate discussions about the consent to blood transfusion, indicating the key issues to be discussed when obtaining informed and valid consent to a blood transfusion, and providing up‐to‐date information on the risks of transfusion. This resource should be provided by the UK Blood Services**.
- **Organisations responsible for training of healthcare staff must include training for consent to blood transfusion in their training curricula**.

## MONITORING COMPLIANCE AND IMPROVEMENT PLANS

The 2024 National Comparative Audits (NCA) of Blood Transfusion found that compliance with the 2016 NICE Quality Standard for Transfusion^6^ was poor: only 36% of patients who had a transfusion had documentation that they were given verbal and written information about blood transfusion. Future NCAs should include consent to blood transfusion to continue to provide compliance data and identify areas for improvement.

**Recommendation**

- **Hospitals should employ mechanisms through their arrangements for clinical governance to monitor the implementation and compliance with these SaBTO recommendations with subsequent improvement plans developed and implemented if necessary**.

## Supporting information


Appendix S1.

Appendix S2.

Appendix S3.

Appendix S4.
