# Supplementary material for: Guidelines from the expert advisory committee on the Safety of Blood, Tissues and Organs (SaBTO) on patient consent and shared decision‐making for blood transfusion
Source: Br J Haematol. 2025 Sep 9;207(6):2314–21. doi: 10.1111/bjh.70075 (PMC12710154; doi:10.1111/bjh.70075)
Supplement: Supplementary file 1 — Appendix S1. [file BJH-207-2314-s001.docx]

**Appendix 1**

**Summary of Recommendations**

- **The purpose of these updated recommendations is to enhance the provision of pertinent information to patients about blood transfusion, ensure an effective process for obtaining patients’ consent to blood transfusion and support shared decision making. The recommendations are pertinent to patients who may be exposed to blood components and blood products in any healthcare setting.**
- **Informed and valid consent to blood transfusion must be obtained for all patients who may, are likely to, or will definitely receive a blood transfusion. This includes where blood transfusion might be needed at some later time, for example during surgery when the patient is incapacitated. An indication that blood transfusion may be required is the collection of a ‘group and save’ or ‘crossmatch’ sample.**
- **Hospitals must have policies that cover the provision of information about blood transfusion and for obtaining patients’ consent to blood transfusion. The policies must include the processes that healthcare staff should follow and how they should be documented.**
- **Consideration must be given whether the blood transfusion is the only available treatment, whether any alternative treatments are available and suitable, and the risks and benefits of those alternatives to blood transfusion. An active discussion must result in shared decision making, allowing the patient to ask their own questions and to raise any concerns that they wish to be addressed before they decide to receive, or refuse, the blood transfusion. Such shared decision-making discussions must be documented in the patient’s clinical record, ideally electronically.**
- **The following framework (adapted from the NICE Blood Transfusion Guideline 2015 NG24 and taking into account the ruling from Montgomery establishing a duty of care to warn of material risks and the patient’s right to make informed treatment decisions) should be used when providing verbal and written information to patients, and their family members or carers (as appropriate):**
- **The reason for the blood transfusion**
- **The benefits of the blood transfusion**
- **The risks of transfusion – short- and long-term, including any additional risks pertinent to long term multi-transfused patients; risks which are specific to the individual patient and which they may consider to be significant**
- **Any blood transfusion needs specific to them including any requirements for special blood components**
- **Any alternatives that are available, and how they might reduce their need for a blood transfusion**
- **The possible consequences of refusing a blood transfusion**
- **The blood transfusion process**
- **That they are no longer eligible to donate blood in the UK**
- **The ability to withdraw consent**
- **That patients are encouraged to ask questions about their treatment and that care is taken to ensure they understand any risks that they may consider to be significant**
- **Patients are entitled to refuse or withdraw their consent to blood transfusion. This must be documented and managed appropriately. If the patient consents to certain blood components and/or products but not others, this must be documented in both the patient’s clinical and blood transfusion laboratory records and must be easily accessible to treating teams.**
- **Patients who have received a blood transfusion and who were not able to give informed and valid consent prior to the blood transfusion must be informed of the blood transfusion in a face-to-face discussion. This must be documented in the patient’s clinical record and in the hospital discharge summary.**
- **Patients undergoing procedures where they were told that they may or may not need a blood transfusion must be informed whether they had a blood transfusion or not. This should be documented in the patient’s clinical record and in the hospital discharge summary.**
- **Hospitals must have policies that cover consent to blood transfusion for neonates, children and young adults and also for the refusal of consent to blood transfusion in both children and adults.**
- **Children and young adults should be involved in the consent to transfusion process according to their maturity and understanding, and age-appropriate information should be provided to support them.**
- **Consultation with hospitals’ legal teams is recommended whenever there are any concerns about the process for consent to blood transfusion in neonates, children and young adults.**
- **The duration of consent must be discussed and agreed with the patient as part of the shared decision-making process. If it is deemed appropriate that consent may span more than one blood transfusion episode, or across the duration of a patient admission period, and this must be documented in the patient’s clinical record.**
- **There is a duty on staff administering a blood transfusion to check that that documentation for consent to blood transfusion is present and valid before commencing the transfusion. Hospitals must facilitate this step by ensuring that documentation for consent to blood transfusion can be easily found in an agreed standard format and location in the patient’s paper or electronic record.**
- **All patients who have received a blood transfusion must be provided with details of the blood transfusion together with information about any adverse events associated with the blood transfusion. Patients must also be informed that they are no longer eligible to donate blood in the UK. All relevant information must be documented in the patient’s clinical record, ideally electronically, and included in their hospital discharge summary to ensure their family doctor is also aware.**
- **Hospitals who already have electronic patient records must ensure the digital documentation of informed and valid patient consent to blood transfusion. Those hospitals who do not already have electronic patient records must ensure the inclusion of the digital documentation of informed and valid patient consent to blood transfusion in their plans for electronic patient records.**
- **The UK Blood Services should continue to provide a standardised source of information for patients who may receive a blood transfusion in the UK.**
- **Training and competency assessment for taking consent to blood transfusion must be included in programmes of training for all relevant healthcare practitioners, and this must be renewed every 3 years.**
- **A centralised UK wide information resource for healthcare practitioners should continue to be provided to support engagement with patients and facilitate discussions about the consent to blood transfusion, indicating the key issues to be discussed when obtaining informed and valid consent to a blood transfusion, and providing up-to-date information on the risks of blood transfusion. This resource should be provided by the UK Blood Services.**
- **Organisations responsible for training of healthcare staff must include training for consent to blood transfusion in their training curricula.**
- **Hospitals should employ mechanisms through their arrangements for clinical governance to monitor the implementation and compliance with these SaBTO recommendations with subsequent improvement plans developed and implemented if necessary.**
