# Supplementary material for: Guidelines from the expert advisory committee on the Safety of Blood, Tissues and Organs (SaBTO) on patient consent and shared decision‐making for blood transfusion
Source: Br J Haematol. 2025 Sep 9;207(6):2314–21. doi: 10.1111/bjh.70075 (PMC12710154; doi:10.1111/bjh.70075)
Supplement: Supplementary file 2 — Appendix S2. [file BJH-207-2314-s002.pptx]

## Slide 1
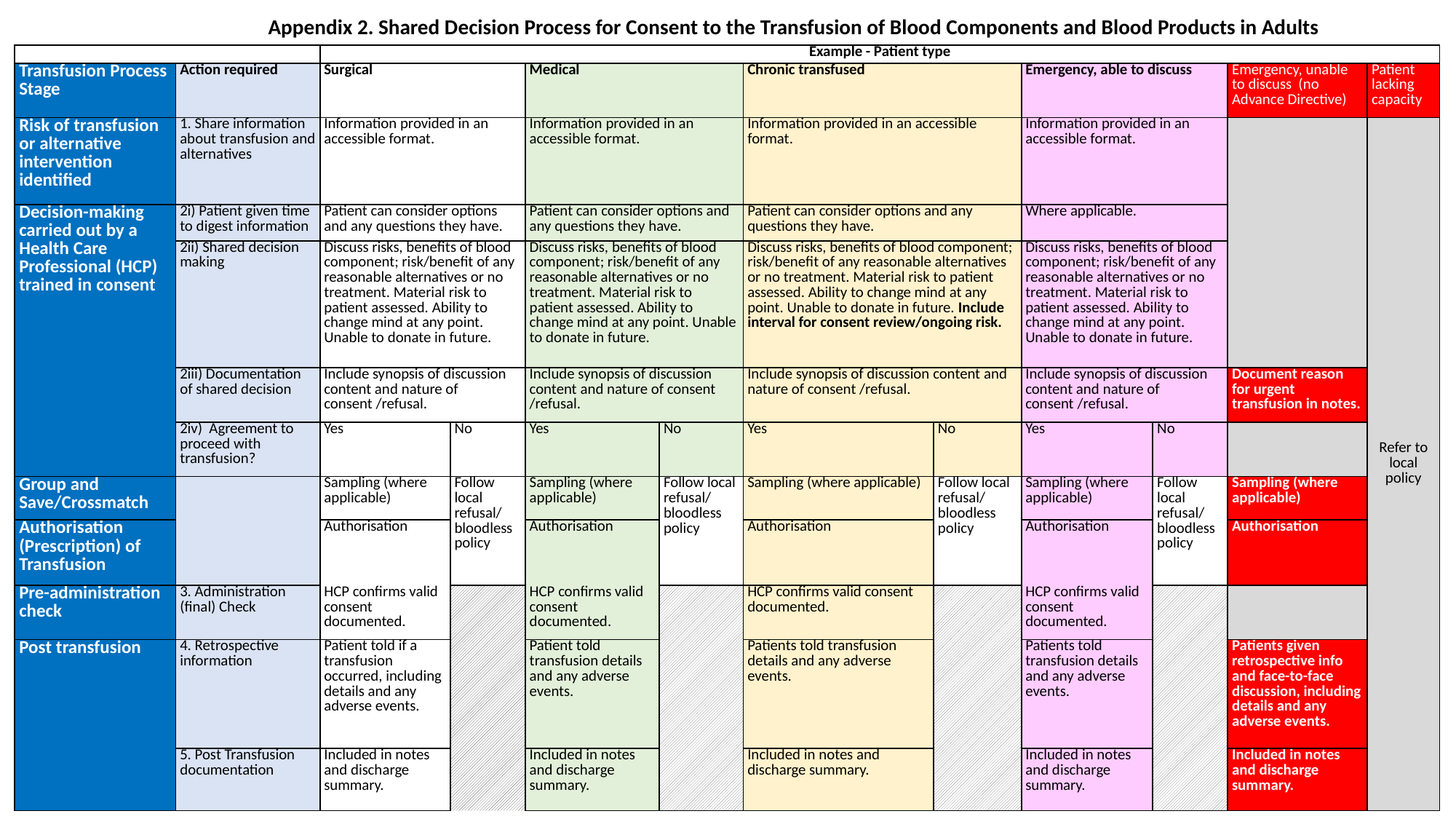

Appendix 2. Shared Decision Process for Consent to the Transfusion of Blood Components and Blood Products in Adults
| | | Example - Patient type | | | | | | | | | |
| --- | --- | --- | --- | --- | --- | --- | --- | --- | --- | --- | --- |
| Transfusion Process Stage | Action required | Surgical | | Medical | | Chronic transfused | | Emergency, able to discuss | | Emergency, unable to discuss (no Advance Directive) | Patient lacking capacity |
| Risk of transfusion or alternative intervention identified | 1. Share information about transfusion and alternatives | Information provided in an accessible format. | | Information provided in an accessible format. | | Information provided in an accessible format. | | Information provided in an accessible format. | | | Refer to local policy |
| Decision-making carried out by a Health Care Professional (HCP) trained in consent | 2i) Patient given time to digest information | Patient can consider options and any questions they have. | | Patient can consider options and any questions they have. | | Patient can consider options and any questions they have. | | Where applicable. | | | |
| | 2ii) Shared decision making | Discuss risks, benefits of blood component; risk/benefit of any reasonable alternatives or no treatment. Material risk to patient assessed. Ability to change mind at any point. Unable to donate in future. | | Discuss risks, benefits of blood component; risk/benefit of any reasonable alternatives or no treatment. Material risk to patient assessed. Ability to change mind at any point. Unable to donate in future. | | Discuss risks, benefits of blood component; risk/benefit of any reasonable alternatives or no treatment. Material risk to patient assessed. Ability to change mind at any point. Unable to donate in future. Include interval for consent review/ongoing risk. | | Discuss risks, benefits of blood component; risk/benefit of any reasonable alternatives or no treatment. Material risk to patient assessed. Ability to change mind at any point. Unable to donate in future. | | | |
| | 2iii) Documentation of shared decision | Include synopsis of discussion content and nature of consent /refusal. | | Include synopsis of discussion content and nature of consent /refusal. | | Include synopsis of discussion content and nature of consent /refusal. | | Include synopsis of discussion content and nature of consent /refusal. | | Document reason for urgent transfusion in notes. | |
| | 2iv) Agreement to proceed with transfusion? | Yes | No | Yes | No | Yes | No | Yes | No | | |
| Group and Save/Crossmatch | | Sampling (where applicable) | Follow local refusal/ bloodless policy | Sampling (where applicable) | Follow local refusal/ bloodless policy | Sampling (where applicable) | Follow local refusal/ bloodless policy | Sampling (where applicable) | Follow local refusal/ bloodless policy | Sampling (where applicable) | |
| Authorisation (Prescription) of Transfusion | | Authorisation | | Authorisation | | Authorisation | | Authorisation | | Authorisation | |
| Pre-administration check | 3. Administration (final) Check | HCP confirms valid consent documented. | | HCP confirms valid consent documented. | | HCP confirms valid consent documented. | | HCP confirms valid consent documented. | | | |
| Post transfusion | 4. Retrospective information | Patient told if a transfusion occurred, including details and any adverse events. | | Patient told transfusion details and any adverse events. | | Patients told transfusion details and any adverse events. | | Patients told transfusion details and any adverse events. | | Patients given retrospective info and face-to-face discussion, including details and any adverse events. | |
| | 5. Post Transfusion documentation | Included in notes and discharge summary. | | Included in notes and discharge summary. | | Included in notes and discharge summary. | | Included in notes and discharge summary. | | Included in notes and discharge summary. | |

## Slide 2
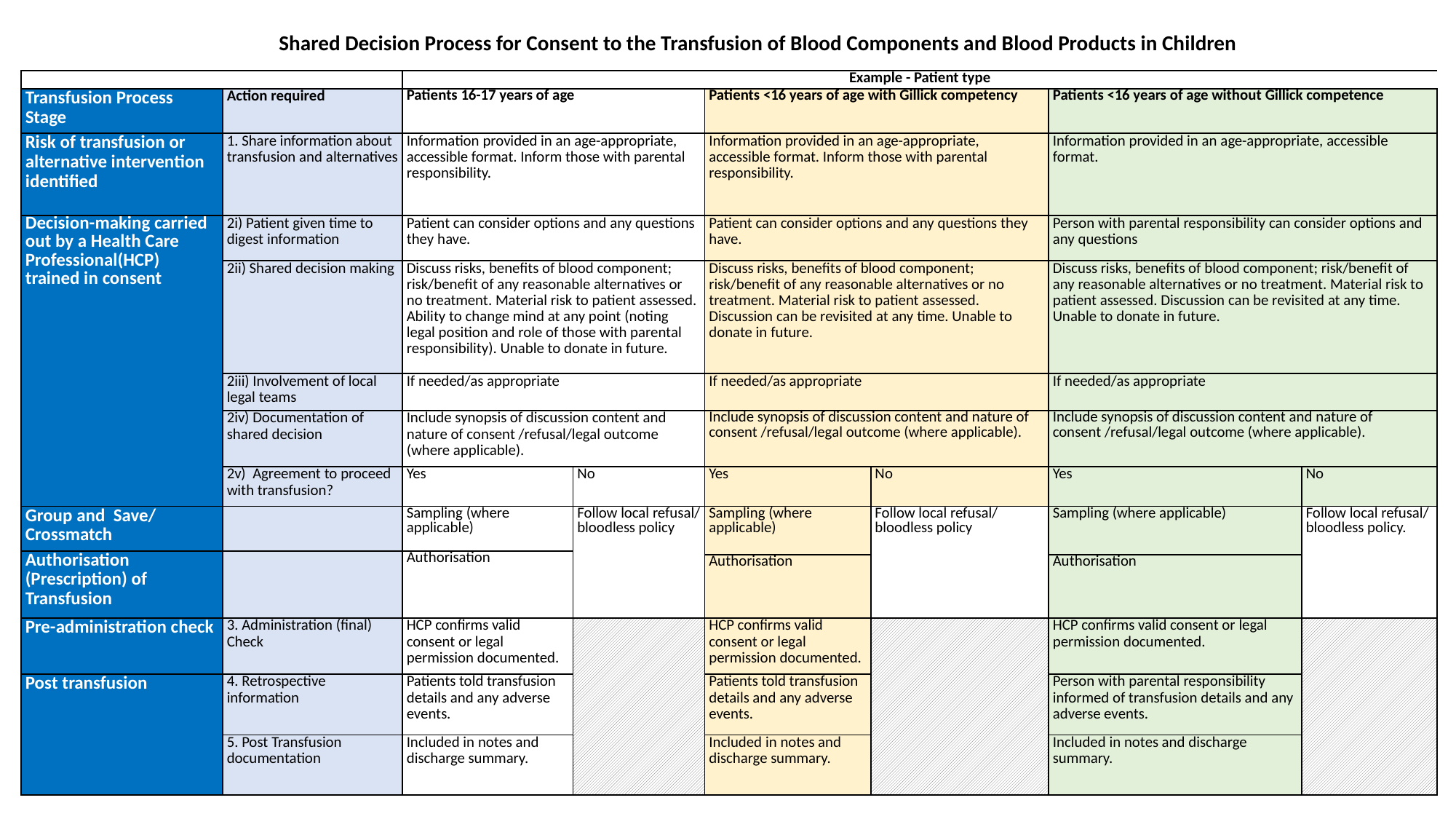

Shared Decision Process for Consent to the Transfusion of Blood Components and Blood Products in Children
| | | Example - Patient type | | | | | |
| --- | --- | --- | --- | --- | --- | --- | --- |
| Transfusion Process Stage | Action required | Patients 16-17 years of age | | Patients <16 years of age with Gillick competency | | Patients <16 years of age without Gillick competence | |
| Risk of transfusion or alternative intervention identified | 1. Share information about transfusion and alternatives | Information provided in an age-appropriate, accessible format. Inform those with parental responsibility. | | Information provided in an age-appropriate, accessible format. Inform those with parental responsibility. | | Information provided in an age-appropriate, accessible format. | |
| Decision-making carried out by a Health Care Professional(HCP) trained in consent | 2i) Patient given time to digest information | Patient can consider options and any questions they have. | | Patient can consider options and any questions they have. | | Person with parental responsibility can consider options and any questions | |
| | 2ii) Shared decision making | Discuss risks, benefits of blood component; risk/benefit of any reasonable alternatives or no treatment. Material risk to patient assessed. Ability to change mind at any point (noting legal position and role of those with parental responsibility). Unable to donate in future. | | Discuss risks, benefits of blood component; risk/benefit of any reasonable alternatives or no treatment. Material risk to patient assessed. Discussion can be revisited at any time. Unable to donate in future. | | Discuss risks, benefits of blood component; risk/benefit of any reasonable alternatives or no treatment. Material risk to patient assessed. Discussion can be revisited at any time. Unable to donate in future. | |
| | 2iii) Involvement of local legal teams | If needed/as appropriate | | If needed/as appropriate | | If needed/as appropriate | |
| | 2iv) Documentation of shared decision | Include synopsis of discussion content and nature of consent /refusal/legal outcome (where applicable). | | Include synopsis of discussion content and nature of consent /refusal/legal outcome (where applicable). | | Include synopsis of discussion content and nature of consent /refusal/legal outcome (where applicable). | |
| | 2v) Agreement to proceed with transfusion? | Yes | No | Yes | No | Yes | No |
| Group and Save/ Crossmatch | | Sampling (where applicable) | Follow local refusal/ bloodless policy | Sampling (where applicable) | Follow local refusal/ bloodless policy | Sampling (where applicable) | Follow local refusal/ bloodless policy. |
| Authorisation (Prescription) of Transfusion | | Authorisation | | | | | |
| | | Authorisation | | Authorisation | | Authorisation | |
| Pre-administration check | 3. Administration (final) Check | HCP confirms valid consent or legal permission documented. | | HCP confirms valid consent or legal permission documented. | | HCP confirms valid consent or legal permission documented. | |
| Post transfusion | 4. Retrospective information | Patients told transfusion details and any adverse events. | | Patients told transfusion details and any adverse events. | | Person with parental responsibility informed of transfusion details and any adverse events. | |
| | 5. Post Transfusion documentation | Included in notes and discharge summary. | | Included in notes and discharge summary. | | Included in notes and discharge summary. | |
