# Supplementary material for: Guidelines from the expert advisory committee on the Safety of Blood, Tissues and Organs (SaBTO) on patient consent and shared decision‐making for blood transfusion
Source: Br J Haematol. 2025 Sep 9;207(6):2314–21. doi: 10.1111/bjh.70075 (PMC12710154; doi:10.1111/bjh.70075)

Appendix 3

Examples of methods to document consent to transfusion from hospitals in the UK.*Documentation of consent for transfusion

- 1. All Wales Transfusion Record. Paper prescription and observation record for transfusion, and a pre-administration checklist including consent for transfusion and assessment for the risk of transfusion-associated circulatory overload (TACO).
  2. Scottish National Blood Transfusion Service. Paper prescription and observation record for transfusion, and a pre-administration checklist including consent for transfusion and assessment for the risk of transfusion-associated circulatory overload (TACO). A flowchart for the management of transfusion reactions is also included.
  3. Northern Ireland. Electronic record and checklist for consent for transfusion.
  4. Oxford University Hospitals, England. Electronic process for consent for transfusion.
  5. Frimley Park Hospitals, England. Electronic process for prescription for transfusion including consent for transfusion.
  6. Hampshire Hospitals, England. Paper record and checklist for consent for transfusion including a section for patients unable to provide consent.
  7. Hampshire Hospitals, England. Paper prescription and observation record for transfusion, and a pre-administration checklist including consent for transfusion.

Documentation of parental consent for transfusion in children

1. Alder Hey Hospital, England. Electronic record and checklist for consent for transfusion.

Documentation of refusal of consent for transfusion

1. Great Ormond Street Hospital, England. Paper record for refusal of consent to transfusion for Jehovah’s Witness patients under the age of 18.
2. Northern Ireland. Electronic record for documentation of Advance Directive and explicit consent or refusal to different blood products.

*Please note that updated versions for these examples might be available now or in the future

Document 1


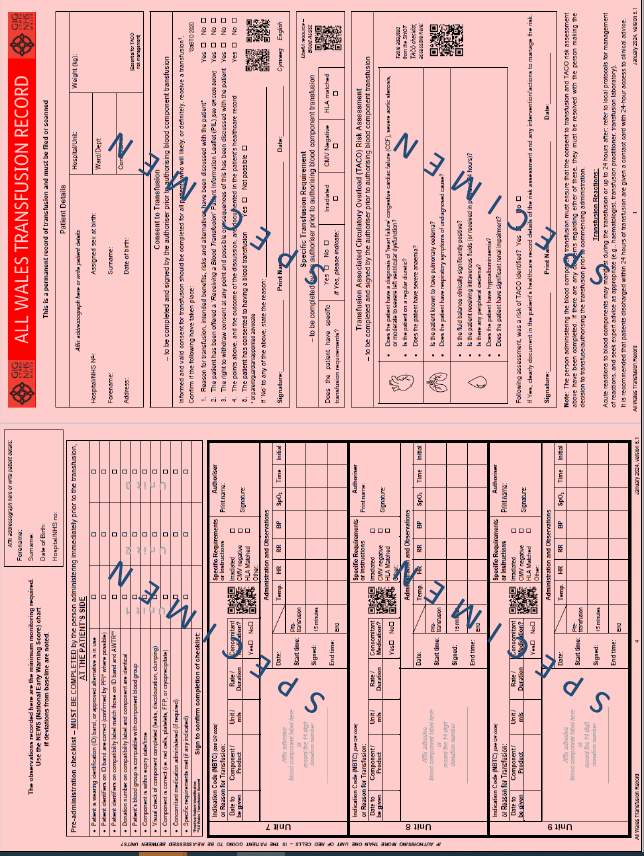


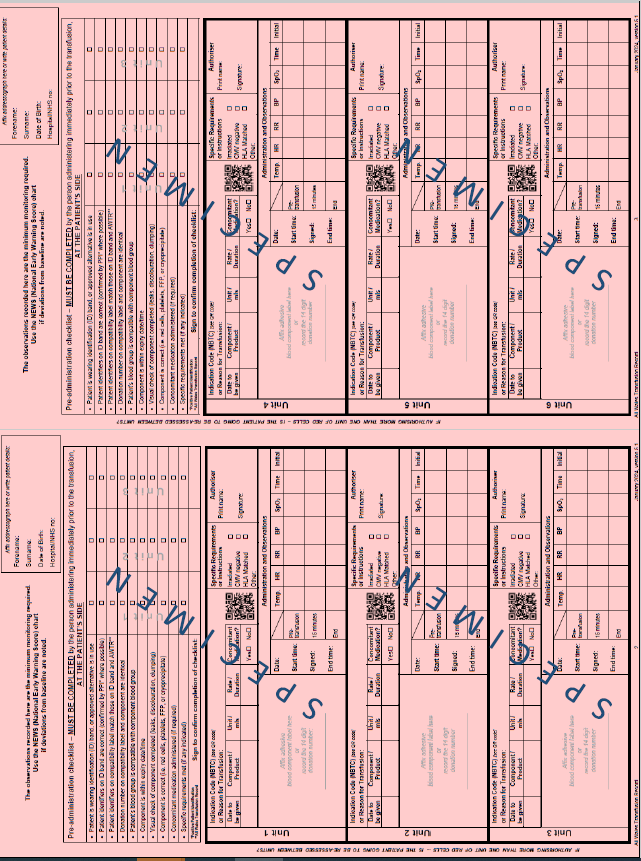
Document 2


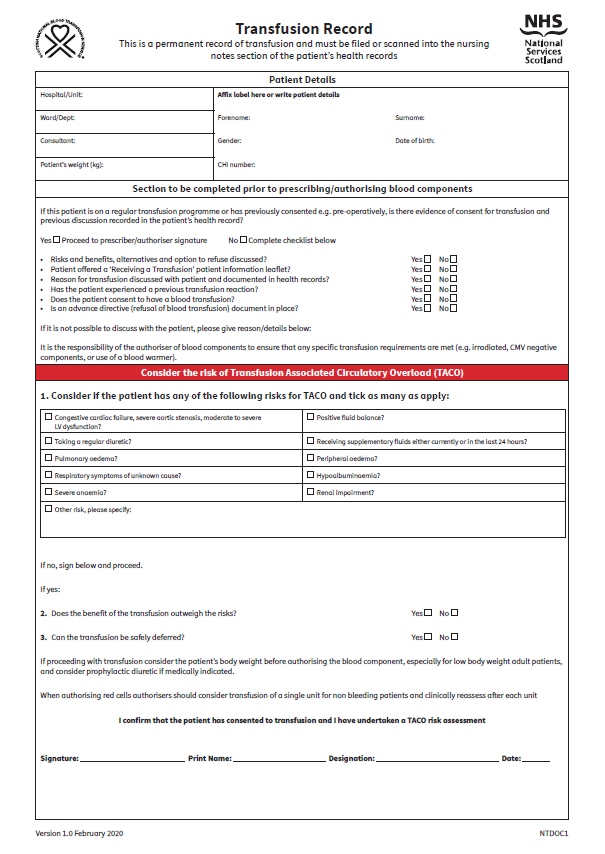


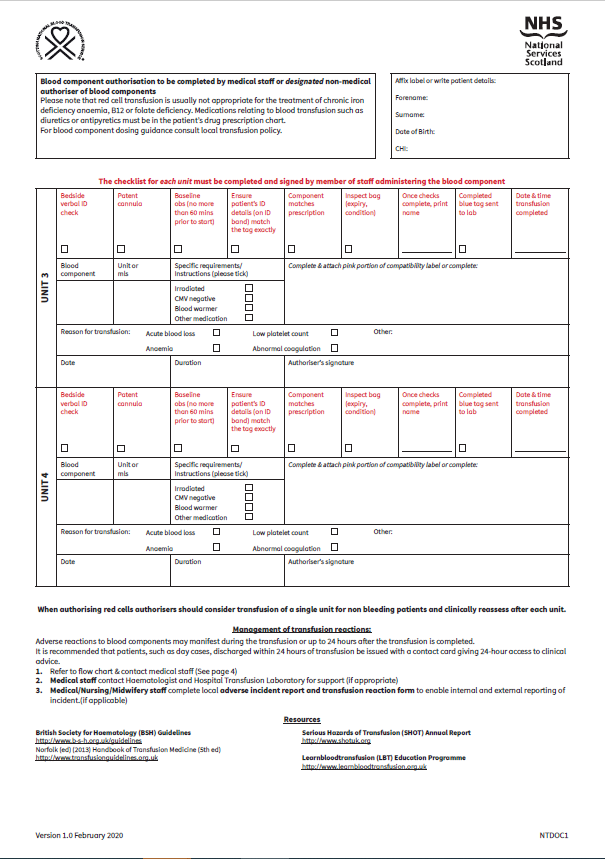

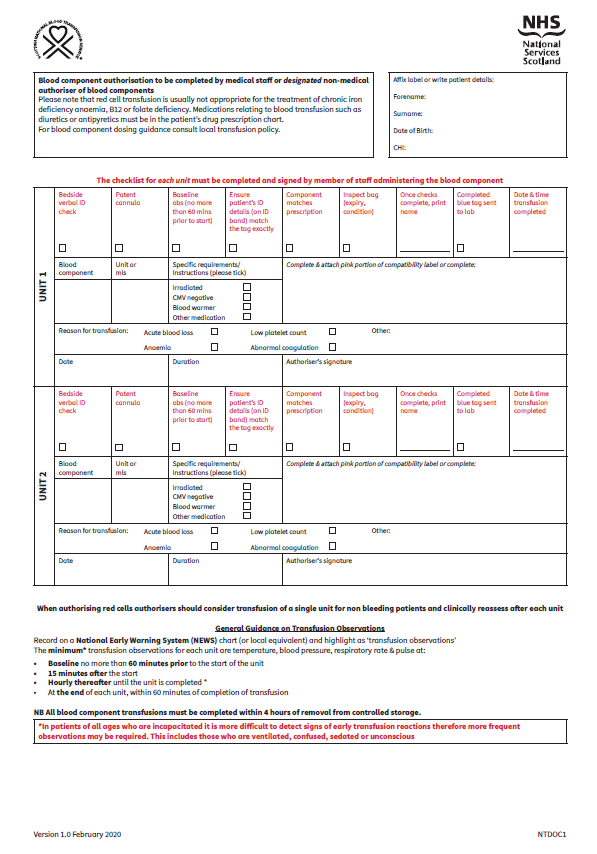


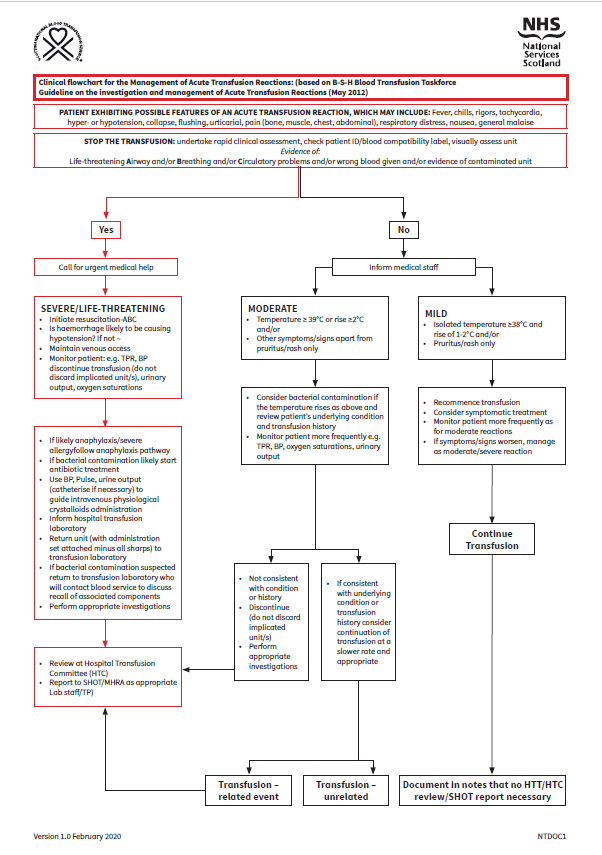


Document 3
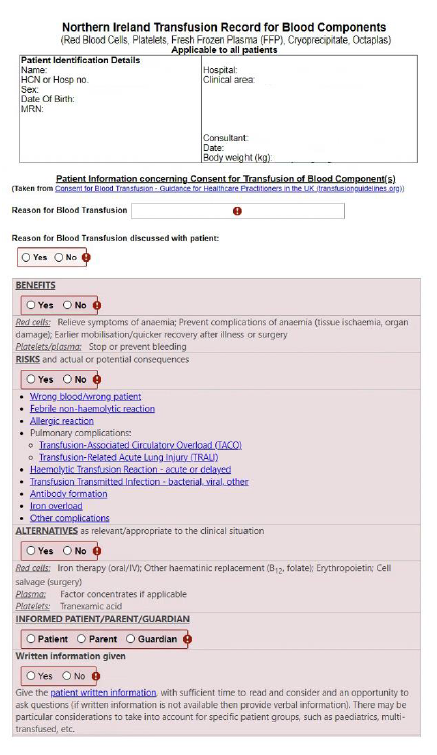


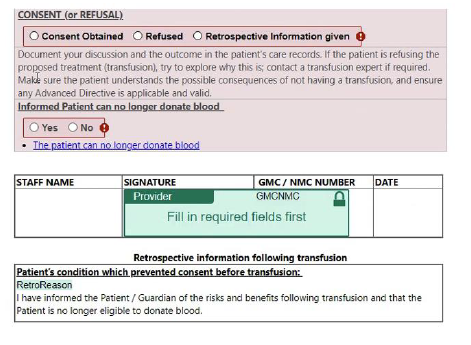


Document 4


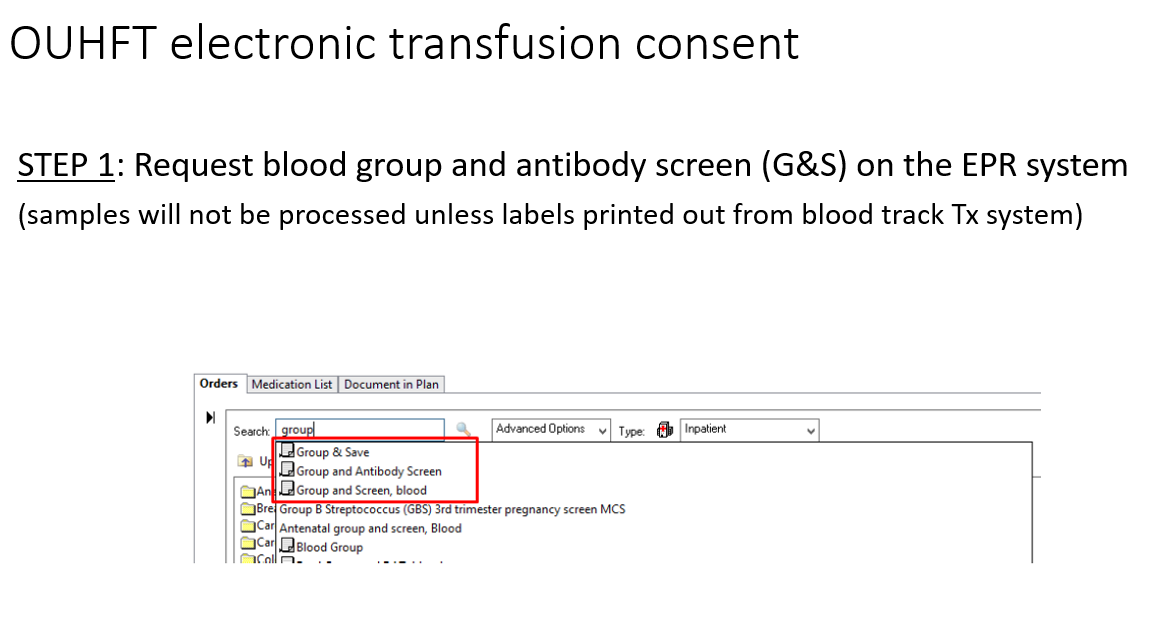


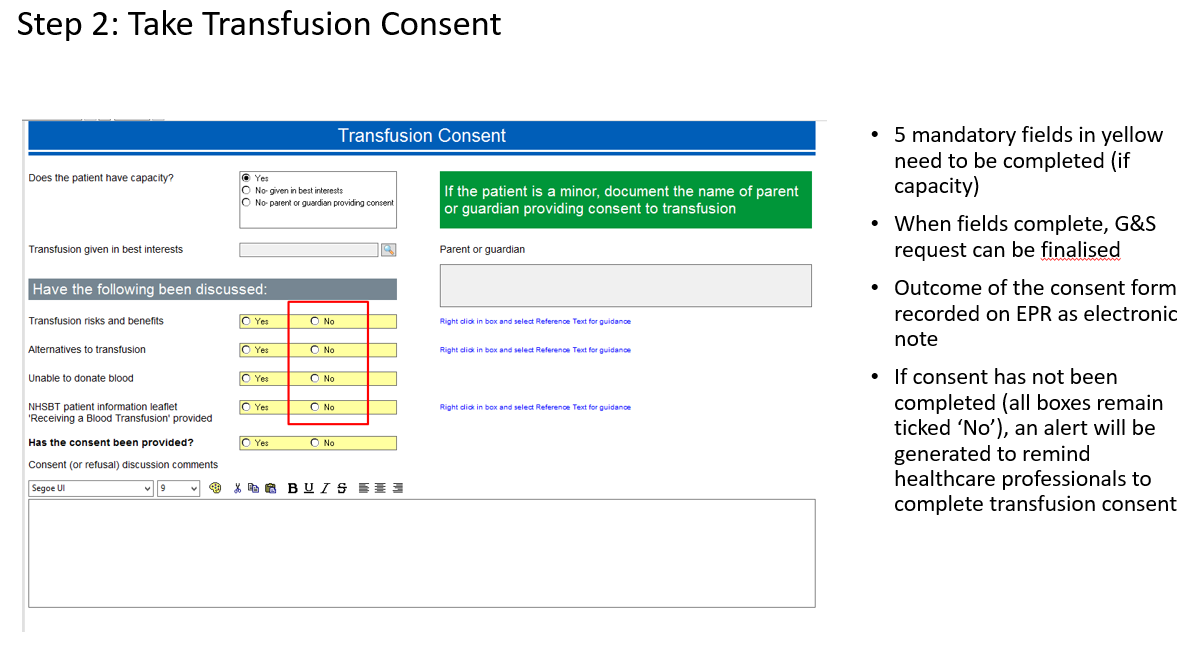


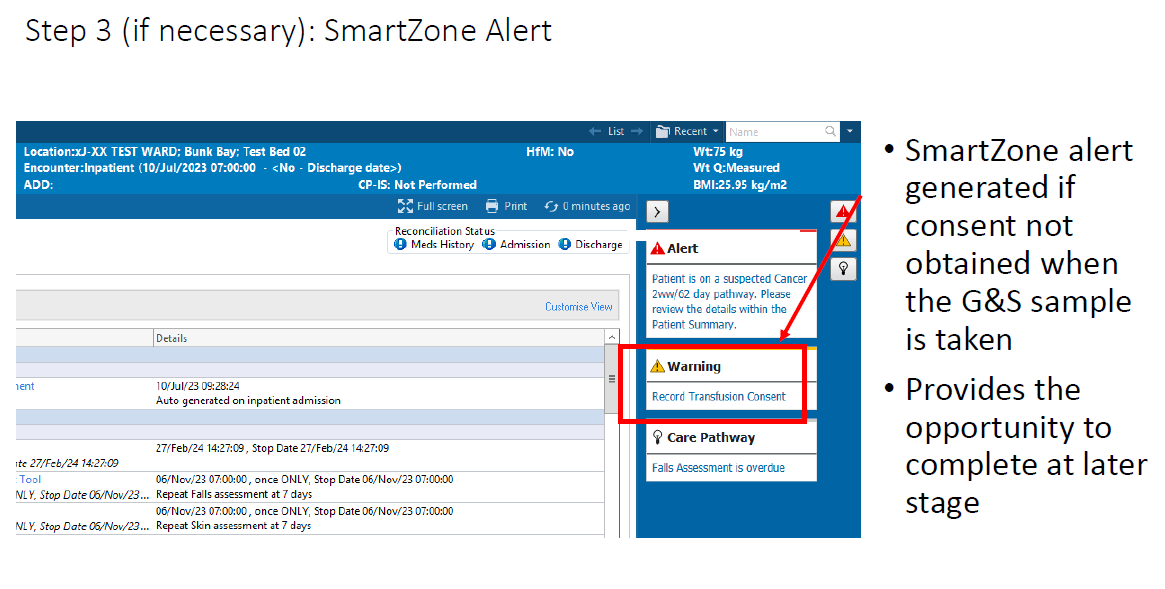


Document 5


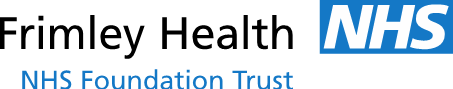


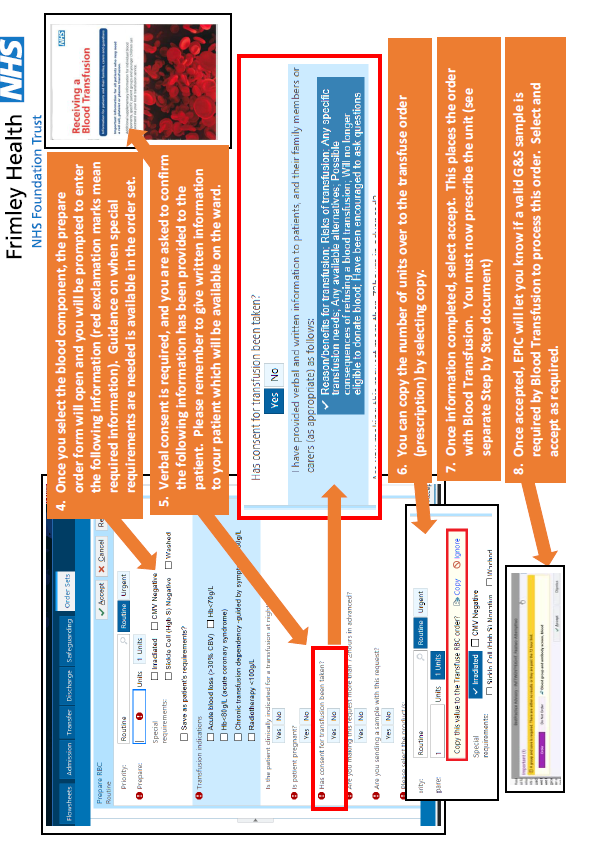


Document 6

Document 7

Document 8


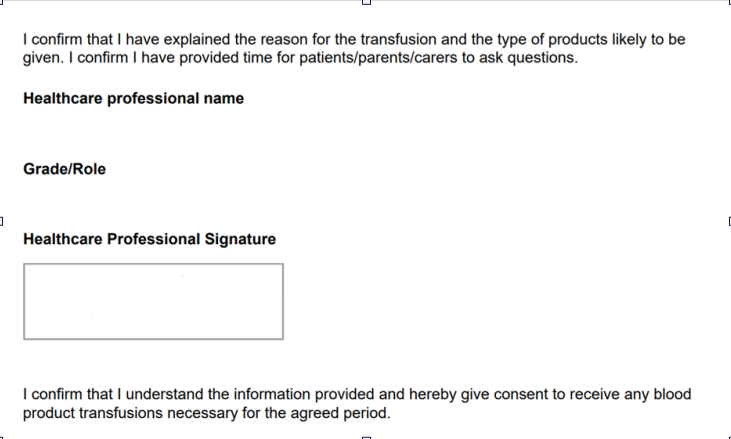

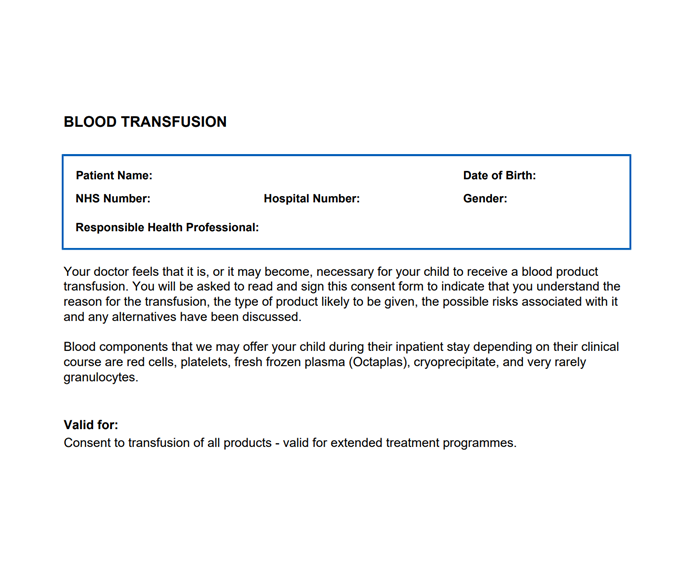


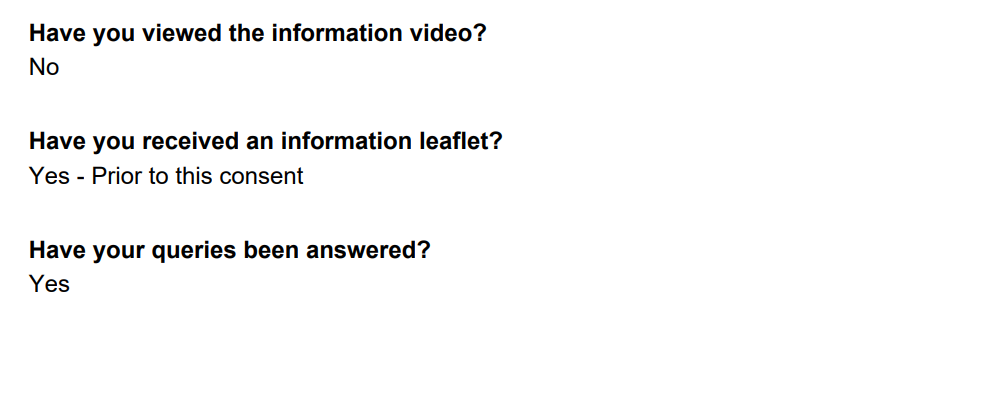


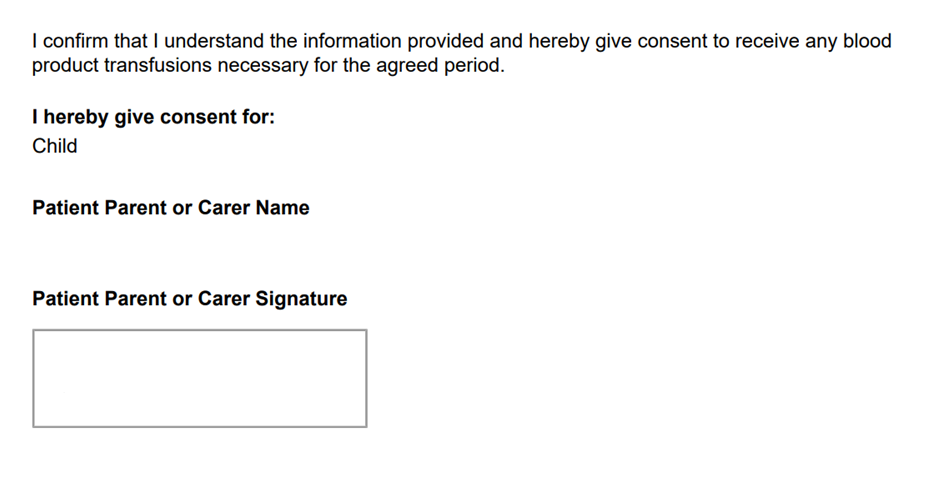


Document 9

| \| N H D \| ame  ospital no OB    Please affix label \| \| --- \| --- \| | 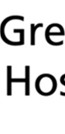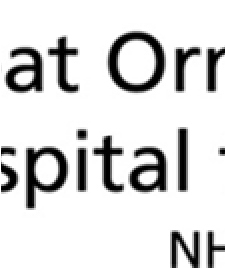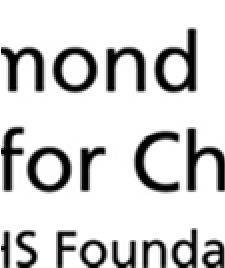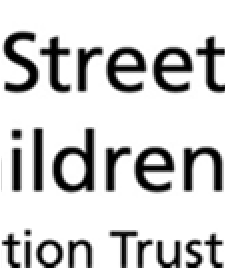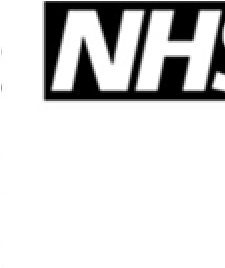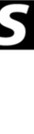 |
| --- | --- | --- | --- |

Additional Consent Form pages for GOSH Jehovah’s Witness patients under the age of 18 years old

***Notes:***

1. ***These two pages are only to be used for GOSH patients under 18 years old.***

1. ***These two pages must be used in addition to the GOSH procedure Consent Forms for (i) under 16 year old patients and (ii) 16/17 year old patients; these should be affixed firmly to the completed Consent Form.***
2. ***These additional two pages are optional. However, any other form from the Jehovah’s Witness Liaison***

***Committee should not be used as a substitute to these additional two pages.***

**Specific procedure(s) to which this form applies:**

# Your views

GOSH acknowledges your wish that no blood products or blood components be given to the patient for this procedure, except those stated **below**.

**List of acceptable blood products and blood components:**

# Emergencies

All GOSH staff will endeavour to avoid use of blood and blood components if this is at all possible without causing risk of death or serious harm to the patient. All possible alternatives to blood, including cell salvage (where possible) and the use of the products or components identified as acceptable to you **above** will be considered first. However, if no alternative is possible in a situation that could lead to death or serious harm and if blood products and blood components are immediately necessary to try to prevent that, then blood products and blood components will be used prior to surgery, during surgery, in the post‐operative period or in an acute medical situation (This will be considered a medical emergency and, in line with the legal position, consent will not be sought for that).

# Non‐emergencies

In relation to situations that do not amount to a medical emergency, and where an application is made for a Court Order, I/we understand that in all cases of which GOSH is aware, the Court in England & Wales has overridden the refusal of blood and blood components where the patient is under 18 years old and provision of blood or blood components is considered by the treating team to be in the patient’s best interests. I/we understand that this is the case even where the patient themselves expresses a clear commitment to the Jehovah’s Witness faith and refuses to accept blood products or blood components for themselves. GOSH is a

hospital that is bound by the law of England & Wales.

ADDITIONAL CONSENT FORM PAGES FOR GOSH JEHOVAH’S WITNESS PATIENTS UNDER 18

© Great Ormond Street Hospital for Children NHS Foundation Trust, 2018

Page **1** of **2**

In recognition of the current legal position in England & Wales, I/we accept the reality that the treating team at GOSH will give blood products and blood components in the following specific circumstances, even when it is not an emergency:

- Where the patient is under 18 years old; **AND**
- Where the provision of blood products or blood components is considered to be in the patient’s best interests – and a member of the clinical team has documented in the medical records the reasons why it is their professional opinion that giving blood products or blood components is in the patient’s best interests; **AND**
- Where in the view of clinical team there is no other clinically appropriate alternative to administering blood or blood components.

Whatever course the treating surgeon and/or the clinical paediatric team follow they will always act in the patient’s best interests, having regard to your known wishes.

These pages will be explained to you/the patient. If you have any further questions please ask – we are here to help you.

I/we understand that I/we have the right to change our mind at any time, including after you have signed these pages.

# Signatures

**Signature of Consultant** (to confirm **ALL** **THREE** bullet points above apply to this patient)

| Signature of Consultant | Date |
| --- | --- |
| Name of Consultant (PRINT) |  |
| Job title |  |
| Contact details of Consultant |  |

**Signature of the under 18 year old patient OR person with Parental Responsibility belonging to the Jehovah’s Witness faith**

***NB: The person signing these additional pages must be the same person who signs the GOSH procedure Consent Form***

| Signature | Date |
| --- | --- |

**Statement of interpreter (where appropriate)**

I have interpreted the information above to the person(s) giving consent to the best of my ability and in a way in which I believe they can understand.

| Name/ID number of interpreter (PRINT) | Date |
| --- | --- |
| Signature of interpreter (if present) |  |
| Signature of health professional (if interpreter not present) |  |

ADDITIONAL CONSENT FORM PAGES FOR GOSH JEHOVAH’S WITNESS PATIENTS UNDER 18

© Great Ormond Street Hospital for Children NHS Foundation Trust, 2018

Page **2** of **2**

Document 10

Patient Interview Prior to Completion Of Advance Directive

It is important to document the information discussed and any additional actions required in the Patient's notes. Patients, including Jehovah's Witnesses often have some knowledge about donated blood components, blood derived products and recombinant coagulation factors, such as recombinant Factor Vila. They may already have made their own personal decisions about which of these treatments they would accept or refuse and whether Or not they would accept cell salvage or cardiopulmonary bypass. However. it is important to clarify the following with the Patient before the Advance Directive is completed:

- Which blood components and products would normally be included in the treatment of major bleeding?
- Treatment options, acceptable to the Patient. which would be available to treat bleeding
- State which treatment options would not be suitable or not available

| Advance Directive for the Consent or Refusal of Blood Components, Blood Products and Transfusion alternatives  For completion by the patient who has reached an informed decision, |
| --- |
| I  Bom on  H&C (or Hospital) no:  Address:  Am Of sound mind and I voluntarily make this Healthcare Advance Directive  It will remain in force for this episode of care or until specifically revoked by me, concerning the following medical treatments: |

- Allow time for the Patient opportunity to discuss the treatment options listed in the Advance Directive with family and Witness Liaison Committee, as appropriate

Contact your Blood Bank about availability Of coagulation factor concentrates, such as Fibrinogen Concentrate and Factor Xlll concentrate.

Completion of Advance Directive

(See "Legal and Ethical Aspects" in Appendix 1 for additional information)

The following Directive should be completed by the Patient, under the supervision Of, and witnessed by a Senior Clinician, i,e, Consultant, Associate Specialist or Staff Grade, The Clinician should verify the following (using open-ended questions, where appropriate):

Patient

Full name :

Date Of birth:

Health and Care or Hospital number

Home address:


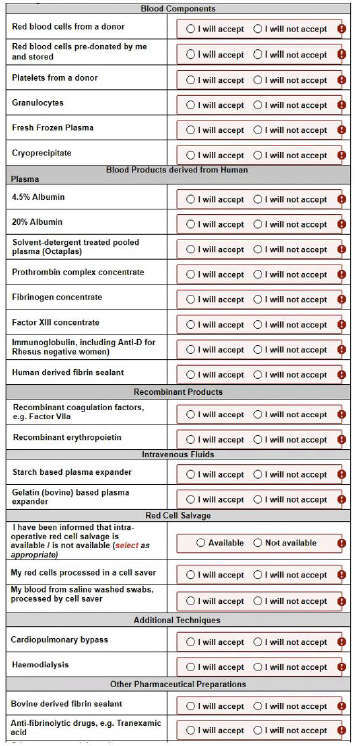


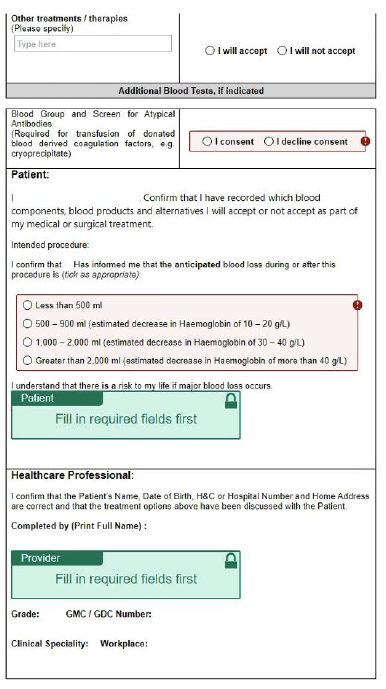

Supplement: Supplementary file 3 — Appendix S3. [file BJH-207-2314-s003.docx]
