# Supplementary material for: Guidelines from the expert advisory committee on the Safety of Blood, Tissues and Organs (SaBTO) on patient consent and shared decision‐making for blood transfusion
Source: Br J Haematol. 2025 Sep 9;207(6):2314–21. doi: 10.1111/bjh.70075 (PMC12710154; doi:10.1111/bjh.70075)
Supplement: Supplementary file 4 — Appendix S4. [file BJH-207-2314-s004.docx]

Appendix 4

Training resources and additional information to support shared decision-making and consent to transfusion

- Animation Resources for consent to transfusion developed by the Scottish National Blood Transfusion Service Transfusion Team

<https://www.nss.nhs.scot/blood-tissues-and-cells/snbts-transfusion-team/resources/>

- Blood Transfusion Training (BTT) e-learning programme for England and Wales (module 3).  This modular training is available for staff involved in all aspects of transfusion.

<https://www.e-lfh.org.uk/programmes/blood-transfusion/>

(Consent to transfusion is covered by an e-learning module as part of the LearnBloodTransfusion (LBT) programme for blood transfusion currently followed in Scotland and Northern Ireland and is available via LearnPro or TURAS Learn)

- Blood Assist App (NHSBT) to assist healthcare practitioners. It was updated in 2024 and includes a section on consent. It can be used on a website or smart phone.

<https://www.bloodassist.co.uk/terms/>

- [Clinical Decision-Making and Authorising Blood Component Transfusion](file:///\\nbscol23\Users\MURP0004\GUIDEL~1\Clinical%20Decision-Making%20and Authorising%20Blood%20Component Transfusion%20https:\transfusionguidelines.org))

An updated document for non-medical authorisation for transfusion was published by the UK and Ireland Blood Transfusion Network in 2022 and includes the framework for appropriately trained and competent healthcare practitioners to assess, consent and authorise transfusion of blood and blood components. practitioners.

[www.transfusionguidelines.org/transfusion-practice/clinical-decision-making-and-authorising-blood-component-transfusion](http://www.transfusionguidelines.org/transfusion-practice/clinical-decision-making-and-authorising-blood-component-transfusion)

- General Medical Council (2024) *Good Medical Practice*.

<https://gmc-uk.org/professional-standards/the-professional-standards/good-medical-practice/>

- General Medical Council (2015) *Promoting Excellence*

[https://www.gmc-uk.org/education/standards-guidance-and-curricula/standards-and-outcomes/promoting-excellence](https://www.gmc-uk.org/education/standards-guidance-and-curricula/standards-and-outcomes/promoting-excellence%20)

- Joint United Kingdom (UK) Blood Transfusion and Tissue Transplantation Services Professional Advisory Committee. Consent for Blood Transfusion - Guidance for Healthcare Practitioners in the UK

[Consent for Blood Transfusion - Guidance for Healthcare Practitioners in the UK](https://www.transfusionguidelines.org/transfusion-practice/consent-for-blood-transfusion/guidance-for-healthcare-practitioners-involved-in-this-role)

- Nursing and Midwifery Council (2018) *The Code*

<https://www.nmc.org.uk/standards/code/>

- Nursing and Midwifery Council (2018) *Realising Professionalism: Standards for Education and Training*

<https://www.nmc.org.uk/standards-for-education-and-training/>
